# Agroecological practices increase farmers’ well-being in an agricultural growth corridor in Tanzania

**DOI:** 10.1007/s13593-022-00789-1

**Published:** 2022-06-16

**Authors:** Sergio G. Milheiras, Susannah M. Sallu, Robin Loveridge, Petro Nnyiti, Lilian Mwanga, Elineema Baraka, Margherita Lala, Eleanor Moore, Deo D. Shirima, Esther N. Kioko, Andrew R. Marshall, Marion Pfeifer

**Affiliations:** 1 School of Natural and Environmental Sciences, Faculty of Science, Agriculture & Engineering, Newcastle University, Newcastle-upon-Tyne, UK; 2 School of Earth and Environment, Faculty of Environment, University of Leeds, Leeds, UK; 3 Department of Environment and Geography, University of York, York, UK; 4 The Biodiversity Consultancy, Cambridge, UK; 5 Reforest Africa, Mang’ula, Kilombero District, Morogoro, Tanzania; 6 Department of Ecosystems and Conservation, Sokoine University of Agriculture, Morogoro, Tanzania; 7 Invertebrate Zoology Section, Department of Zoology, National Museums of Kenya, Nairobi, Kenya; 8 Tropical Forests and People Research Centre, University of the Sunshine Coast, Sunshine Coast, Australia; 9 Flamingo Land Ltd., Malton, UK

**Keywords:** Agroecology, Sustainable agriculture, Land use management, Nature, People, Socioecological systems, East Africa

## Abstract

Millions of people rely on nature-rich farming systems for their subsistence and income. The contributions of nature to these systems are varied and key to their sustainability in the long term. Yet, agricultural stakeholders are often unaware or undervalue the relevance of those contributions, which can affect decisions concerning land management. There is limited knowledge on how farming practices and especially those that build more strongly on nature, including agroecological practices, may shape farmers’ livelihoods and well-being. We aim to determine the effect that farmer perception of contributions from nature, socioeconomic conditions, and farming practices, have on outcomes related to food security and human well-being. We conducted 467 household surveys in an agricultural growth corridor in rural Tanzania, which is also essential for nature conservation due to its high biodiversity and its strategic location between several protected areas encompassing wetland, forest, and grassland habitats. Results show that implementing more agroecological practices at farm scale has a positive effect on farmer well-being in the study landscape. Results also indicate that higher awareness of benefits from nature, as well as engagement with agricultural extension services, are associated with higher number of agroecological practices applied in the farm. This research confirms the relevance of capacity-building initiatives to scale up the uptake of agroecological practices in the tropics. It also shows, using empirical evidence, that farming practices taking advantage of nature’s contributions to people can positively affect food security and human well-being, even when those practices complement conventional ones, such as the use of synthetic inputs. Understanding the impact of agroecological farming on the well-being of smallholder farmers in the tropics paves the way for policy and program development that ensures global food demands are met in a sustainable way without compromising the well-being of some of the world’s most vulnerable people.

## Introduction

One of the greatest challenges of our time is how to improve agricultural practices to meet increasing global food demand in a sustainable way (Van Ittersum et al. 2016; FAO 2017). Industrialized or conventional approaches to crop production are favored by current policy and market conditions. However, there are growing concerns about their long-term sustainability and pressure on planetary boundaries (Campbell et al. 2017; Kremen and Merenlender 2018). Agroecological practices, proposed as alternatives, can help accomplish a transition towards more sustainable food systems (Caron et al. 2014; HLPE 2019). Agroecological practices are defined as agricultural practices aiming to produce significant amounts of food, which integrate ecological processes and ecosystem services (Wezel et al. 2014). Its principles include nutrient recycling, enhancement of soil health, reduction of external inputs, and biodiversity conservation (Wezel et al. 2020). Both the principles and practices of agroecology are sometimes blurred with other concepts, such as ecological intensification (Wezel et al. 2015). Likewise, agroecological intensification is to “improve the performance of agriculture while minimizing environmental impacts and reducing dependency on external inputs through integration of ecological principles” (Wezel et al. 2015). Aligning with the same common principles, in this study we consider agroecological intensification as the application of multiple practices, namely, rainwater harvesting and storage, water retaining pits, fodder banks, cover cropping, mulching, crop rotation, reduced tillage, no tillage, intercropping, post-harvest use of residues, manuring, integrated soil management, fallow, use of natural predators, natural pesticides, and agroforestry (Wezel et al. 2015; HLPE 2019).

Natural elements, including biodiversity and ecological interactions, play a fundamental role in agroecological practices, as they underpin the trade-offs and synergies occurring between multiple ecosystem services (e.g., pollination) and disservices (e.g., crop pests) at farm or landscape scale (Zhang et al. 2007). Within agricultural landscapes in the tropics, agroecological practices can enhance food security, safety, and livelihood outcomes for smallholder farmers and increase the long-term sustainability and resilience of those systems, including retention of its biodiversity (Chappell and LaValle 2009; Duriaux Chavarría et al. 2018; Mdee et al. 2018; HLPE 2019). It has been shown that farming systems integrating agroecological practices have the potential to improve indicators of household well-being, including dietary diversity and nutrition, through subsistence and income-generating pathways, while significantly reducing the negative externalities of farming (Kremen and Miles 2012; Jones 2017; Mdee et al. 2018). For example, a long-term study focused on an agroforestry program in Kenya found positive effects of that intervention on household asset accumulation, particularly in female-led households, fuelwood access, and income generation (Hughes et al. 2020). Nevertheless, smallholder farmers and other relevant stakeholders may be unaware or undervalue the benefits they receive from working with nature (Kleijn et al. 2019). The cross-cutting feature of agroecological practice in the context of rural development creates potential to contribute to the achievement of multiple Sustainable Development Goals, including reducing poverty (SDG1), ensuring conservation, restoration and sustainable use of land (SDG15), improving water quality (SDG6), improving good health and well-being (SDG3), reducing inequalities (SDG10), responsible consumption and production (SDG12), and climate action (SDG13) (Mbow et al. 2014; HLPE 2019). This is particularly relevant when many of the world’s most vulnerable people are smallholder farmers in the tropics (Morton 2007).

Agricultural growth corridors have been established across Africa to promote agricultural development and the closure of yield gaps (Enns 2018). Initiatives so far predominantly used conventional approaches to agricultural intensification, including intensified application of synthetic inputs and conversion of natural habitats to cropland, which carry both environmental and social risks (Pretty and Bharucha 2014; Laurance et al. 2015). When those risks are taken into account in decision-making processes, the areas identified as most suitable to development can change (Laurance et al. 2015; Nijbroek and Andelman 2016). If initiatives fitted for the needs of large agribusinesses exacerbate imbalances in social equity and local access to land and markets, smallholders are unlikely to benefit, with benefits being captured instead by local elites (Sulle 2017). For agricultural development corridors to overcome inconsistencies in their win-win narratives, nuanced perspectives are required, supported by empirical data to help policymakers better address the social and environmental changes caused by corridor routes (Enns 2018). Moving corridor planning towards land management interventions that promote and take advantage of the benefits from agrobiodiversity, such as agroecological practices, allows achieving higher multifunctionality in landscapes that work both for people and nature (Kremen and Merenlender 2018).

Human well-being is vital for our physical, social, psychological, and spiritual fulfilment (MEA 2003). There are still significant knowledge gaps concerning the extent that land use management, including biodiversity conservation or sustainable farming practices, impact smallholder farmers’ livelihoods and well-being (Caron et al. 2014; Milner-Gulland et al. 2014). It is known that biodiversity is of central importance to human well-being and intrinsically linked to sustainable development (MEA 2003; Naeem et al. 2016; Soga and Gaston 2016). Furthermore, there is evidence suggesting that economic outputs at farm level are not sufficient to understand farmer well-being in rural communities (Rivera et al. 2018). As well as a growing recognition of the need to shift measurement of development away from simplistic economic metrics to human well-being (Stiglitz et al. 2009; Dasgupta 2021). That questions the rationale for agricultural growth corridors focused solely on conventional intensification. Recent advancements in the field have increased our ability to capture the multidimensional scope of well-being in an objective metric and use it to assess the outcomes of agricultural interventions in a more comprehensive way (Agarwala et al. 2014; Costanza et al. 2016; Rasmussen et al. 2018; Beauchamp et al. 2018; Loveridge et al. 2020).

**Fig. 1:**
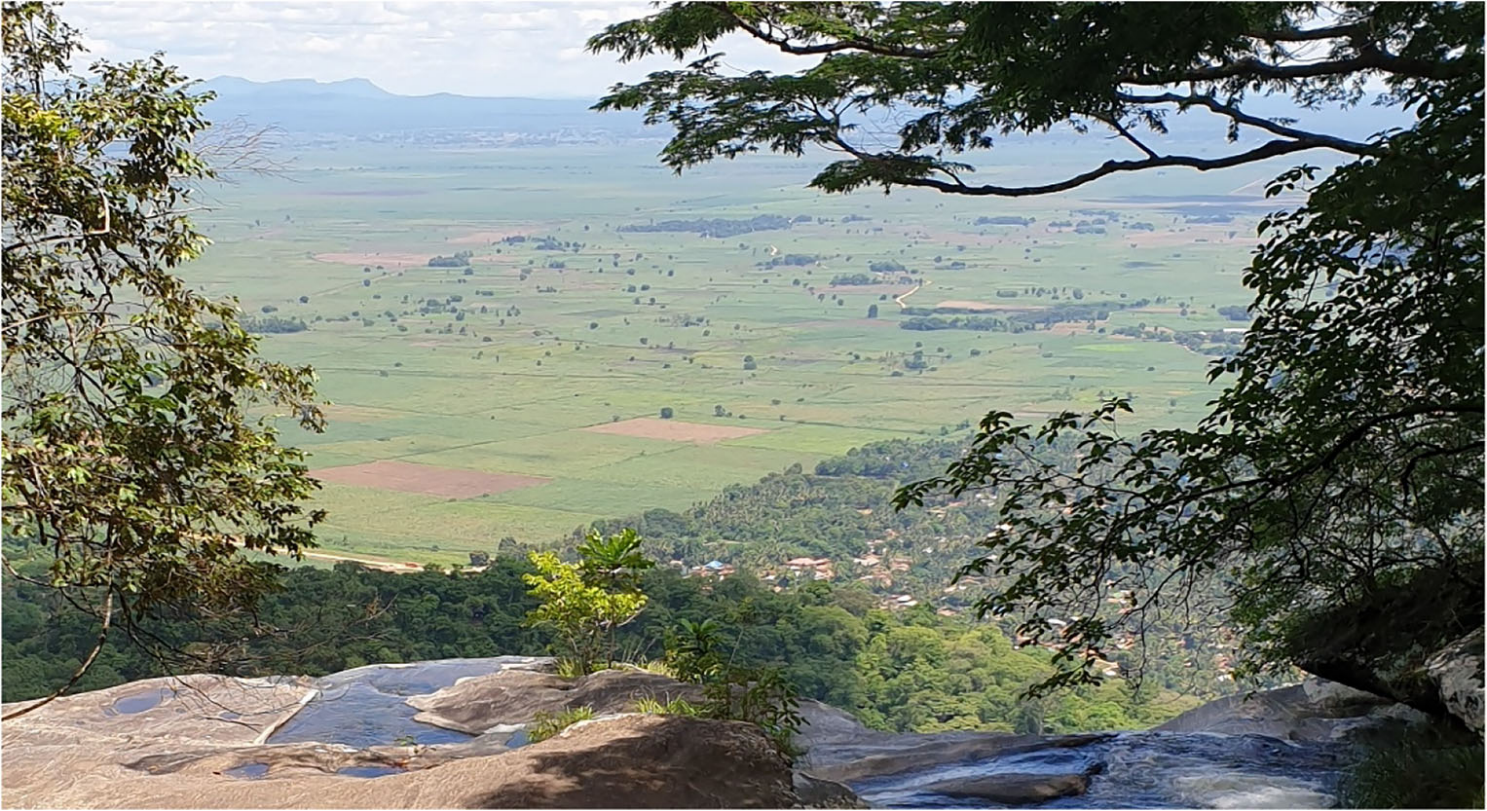
A nature-rich farming landscape in northern Kilombero, Tanzania. The well-being of smallholder farmers in this landscape is interlinked with multiple environmental and socioeconomic factors. Photograph by the authors

In this study, we have the unique opportunity to use household data from a case study in Tanzania to investigate relationships between nature, food security, and human well-being. We use an empirical approach to explore how smallholder farmer perceptions of contributions from nature, socioeconomic conditions, and farming practices, interlink with agroecological intensity, food security, and human well-being, in the context of rural landscapes in the tropics (Fig. 1). More specifically, our research objectives are to determine whether (i) farmer socioeconomic conditions and their perceptions of the contributions from nature drive the uptake of agroecological practices; (ii) higher farm-level agroecological intensity has an impact on the perceived yield of staple crops; and (iii) higher farm-level agroecological intensity contributes to farmer well-being.

## Methods

### Study area

**Fig. 2:**
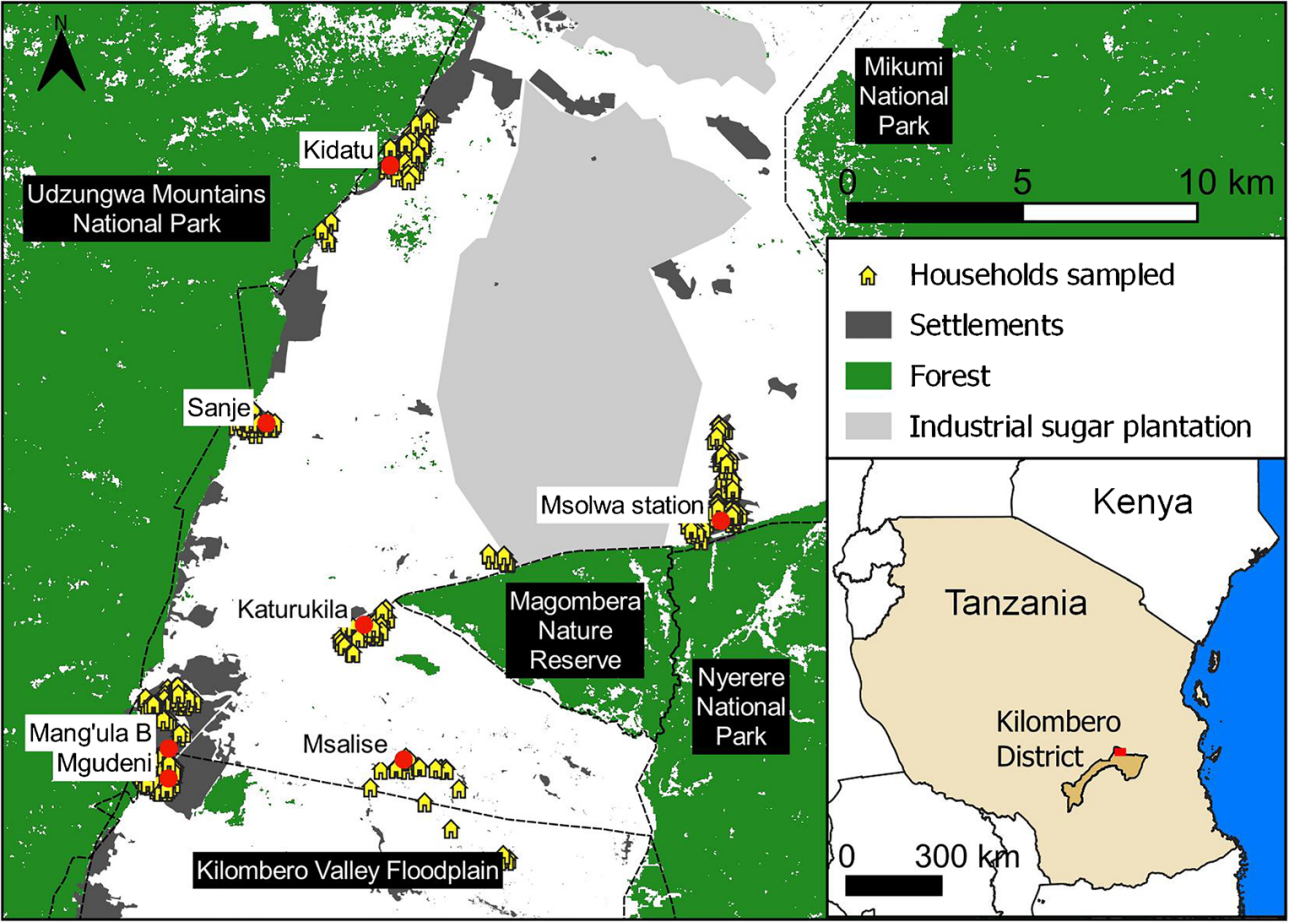
Location of sampled households within the study area. We sampled seven villages (red circles), namely, Kidatu, Msolwa Station, Sanje, Katurukila, Mang’ula B, Mgudeni, and Msalise. The inset map positions the study area (small red rectangle) within Tanzania, East Africa

We conducted this study in the northern part of Kilombero District, Morogoro Region, Tanzania (Fig. 2). Smallholder farming, particularly maize, rice, and sugarcane, and a large commercial sugarcane farm are major land uses in that area. The District constitutes the Kilombero cluster of the Southern Agricultural Growth Corridor of Tanzania (SAGCOT), a priority area for agricultural development (SAGCOT 2011). SAGCOT plans for that cluster include a 320-km road upgrade, 60-km power transmission lines, several thousand hectares of land converted to large commercial farming, and promotion of links between agribusiness and smallholder farmers via out-grower schemes (SAGCOT 2011). Kilombero District also constitutes part of a floodplain that was designated a Ramsar Site in 2002 and is one of the largest wetlands in Africa (Dinesen 2016). The area is important for wildlife mobility—including large mammals—and habitat connectivity, due to its strategic location between several different protected areas. Within the north of Kilombero District, we randomly selected seven villages with a total of 38,456 inhabitants (National Bureau of Statistics 2013) (Fig. 2).

### Data collection

We conducted a survey of 467 randomly selected households in seven villages from four wards in northern Kilombero District (Fig. 2). Sampling effort was proportional to village population size (or sub-village when data was locally available). Data collection occurred between November and December 2019. We randomly selected households after consulting the respective village registries to ensure that our sample reflected local socio-economic variation. Only one individual was surveyed per household, since well-being is highly correlated between members of the same household (de Lange et al. 2016). To avoid gender bias, men and women were selected in rotation from one household to the next, whenever possible. Three locally hired enumerators with previous experience were trained and applied the surveys. A pilot study and focus group discussions were conducted in two of the sampled villages to guarantee the wording was adapted to the local context. The questionnaire was developed in English and translated to Swahili using clear and simple language. For data entry, we used Open Data Kit (ODK) tools, specifically ODK Collect and Aggregate (Hartung et al. 2010). Free, prior, and informed consent was obtained before starting the questionnaire. The questionnaire aimed to gather information on farmers’ agricultural practices, their interaction with nature, and their well-being levels. It took approximately 60 min to complete and consisted of nine different sections: basic information, household characteristics, health and education, livelihood and labour, assets, perspectives on ecosystem services and disservices, perspectives on nature, quality of life, and farming practices. Its design integrated elements from previous works developed in Tanzania (CGIAR (Consultative Group on International Agricultural Research) 2015; EDI (Economic Development Initiatives) 2007; Loveridge et al. 2020; World Bank 2016). For the map in Fig. 2, the forest layer was developed by the European Space Agency using Sentinel-2A data (ESA, 2017). Dwelling areas were classified manually. Spatial data for the protected areas represented, including Kilombero Valley Floodplain Ramsar site and Nyerere National Park (previously Selous Game Reserve), was downloaded from the World Database on Protected Areas (UNEP-WCMC and IUCN, 2021).

### Modelling approach

To investigate how smallholder farmers’ socioeconomic conditions, their perceptions, and farming practices relate with food security and human well-being, we used a three-tiered modelling approach. This followed our understanding of causal pathways starting at farmer perceptions, socioeconomic conditions, and farming practices and leading to (i) agroecological intensity, (ii) food security, and finally to (iii) well-being. The systems approach framework underlying relationships within the causal pathways was described in Milheiras et al. (2022). We analyzed pathways using three different models with agroecological intensity (model 1), estimated crop yield (model 2), and human well-being (model 3) as response variables. Considering that our focus are smallholder farmers in tropical countries, we worked under the assumption that crop yield is highly correlated with food security for this context (IFAD and UNEP 2013).

We also assumed that agroecological intensity, food security, and human well-being can be affected by the same socioecological factors. This builds on existing literature suggesting that perceptions of nature impact on uptake of sustainable farming practices (Piñeiro et al. 2020), food security (Akinnifesi et al. 2010), and well-being (Hartig et al. 2014), and so do socioeconomic conditions (Bashir and Schilizzi 2013; Kassie et al. 2013; Reyes-García et al. 2016). There is less evidence on the effects that agroecology uptake has on food security (Altieri et al. 2012) or on well-being (Miller et al. 2020; Ojedokun et al. 2020). However, the association between food security and human well-being is well established (Frongillo et al. 2017).

The variables used in our three-tiered modelling approach were selected after checking for multicollinearity and missing values. We excluded as predictors variables already being used to generate the well-being composite indicator. The description of all model covariates can be found in Table 1. The description of well-being indicators can be found in Table 2.

**Table 1:** Description of the model covariates used in models 1, 2, and 3. The response variables were agroecological intensity (model 1), staple crop yield (model 2), and human well-being (model 3)

Variable	Description | variable type | categories	Mean (SD)	Range
Age	Respondent’s age | Integer	49.96 (14.05)	20–96
Gender	Respondent’s gender | Binary | ‘1’= woman, ‘0’= man	0.52 (0.5)	0–1
Village responsibilities	Has local role or responsibility for which respondent is publicly known | Binary | ‘1’= yes, ‘0’= no	0.21 (0.41)	0–1
Group membership	Number of local groups or associations the respondent is member of | Integer	1.2 (1.34)	0–7
Food needs	Frequency of difficulties satisfying the food needs of the household | Ordinal | ‘4’= Never, ‘0’= Always	2.6 (1.22)	0–4
Perceived crop damage	Lost more than a ¼ crop production to pests and/or mammals in the last year | Binary | ‘1’= yes, ‘0’= no	0.62 (0.49)	0–1
Perceived ecosystem services	Total number of ecosystem services listed as being provided by natural habitats in and around the farm | Integer	5.2 (3.4)	0–22
Perceived nature impact on livelihood	Perceived overall impact of natural areas on and around the farm on the respondent’s livelihood | Ordinal | ‘5’= very good, ‘1’= very bad	3.84 (1.21)	1–5
Perceived future conditions	How respondent believes natural environmental will be in 5 years | Ordinal | ‘3’= better than now, ‘1’= worse than now	2.03 (0.79)	1–3
Farming advice	Farmer received farming advice in the last 3 years | Binary | ‘1’= yes, ‘0’= no	0.3 (0.46)	0–1
Plot ownership	If respondent’s household owns farm plots | Binary | ‘1’= yes, ‘0’= no	0.69 (0.46)	0–1
Synthetic inputs	Number of different synthetic inputs (inorganic fertilizer, pesticides, herbicides, fungicides) used at farm-scale | Integer	1.69 (1.15)	0–4
Agroecological intensity	Number of different agroecological practices used at farm-scale | Integer	1.46 (1.8)	0–12
Staple crop yield	Estimated productivity (kg/acre) of maize and rice in last year, both normalized on a 0-1 scale and added together | Interval	0.13 (0.17)	0–1.06
Well-being	Composite indicator of human well-being calculated from 20 indicators | Interval	0.55 (0.17)	0.12–1

**Table 2:** Indicators used to calculate the well-being composite index. The indicators were selected based on the methodology developed by Loveridge et al. (2020). All variables were normalized prior to the calculation

Variable	Description | variable type | categories	Mean (SD)	Range
Material			
Financial savings	Has financial savings | Binary | ‘1’= yes, ‘0’= no	0.21 (0.41)	0–1
Household wall material	Material used for household walls | Ordinal | ‘3’= concrete bricks, ‘2’= plastered mud bricks, ‘1’= mud bricks, ‘0’= mud and sticks	1.27 (0.55)	0–3
Household assets	Total of assets owned in a list of 13 household items | Integer	4.33 (2.11)	0–10
Banking	Uses formal banking services | Binary | ‘1’= yes, ‘0’= no	0.63 (0.48)	0–1
Water access	Walking time (minutes) to reach drinking water supply | Ordinal | ‘2’= [0-1], ‘1’= ]1-10[, ‘0’= [10-120]	1.29 (0.67)	0–2
Land area	Total farm area owned (acres) | Ordinal | ‘4’= >10, ‘3’= ]5, 10], ‘2’= ]2, 5], ‘1’= ]0,2], ‘0’= none	1.54 (1.37)	0–4
Livestock	Most valuable livestock owned | Ordinal | ‘3’= cattle, ‘2’= pigs, sheep, goat, ‘1’= poultry, fish, rabbits, ‘0’= none	0.66 (0.64)	0–3
Health			
Sickness	Too unwell to work in the last year | Binary | ‘1’= no, ‘0’= yes	0.39 (0.49)	0–1
Health insurance	Has health insurance | Binary | ‘1’= yes, ‘0’= no	0.17 (0.38)	0–1
Diet diversity	Number of different food items eaten in last 7 days | Integer	8.05 (2.21)	2–12
Social relations			
Borrowing of resources	Borrowed money in last year including informal loans | Binary | ‘1’= yes, ‘0’= no	0.4 (0.49)	0–1
Recognition in the village	Perception that voice is heard in important village decisions | Ordinal | ‘2’= yes, ‘1’= don’t know, ‘0’= no	1.4 (0.74)	0–2
Security			
Provision for dependents	Confidence in providing for dependents | Ordinal | ‘4’= very confident, ‘3’= somewhat confident, ‘2’= neutral/ don't know, ‘1’= somewhat uncertain, ‘0’= very uncertain	2.37 (1.38)	0–4
Provision for self in old age	Confidence in providing for oneself in old age | Ordinal | ‘4’= very confident, ‘3’= somewhat confident, ‘2’= neutral/ don't know, ‘1’= somewhat uncertain, ‘0’= very uncertain	2.12 (1.3)	0–4
Number of livelihoods	Total of different livelihood-generating activities | Integer	4.22 (2.02)	1–11
Theft security	Perception of security from theft | Ordinal | '4'= very safe, '3'= somewhat safe, '2'= neutral/ don't know, '1'= somewhat unsafe, '0'= very unsafe	1.97 (1.31)	0–4
Freedom			
Livelihood satisfaction	Satisfaction with livelihood opportunities | Ordinal | '4'= very satisfied, '3'= somewhat satisfied, '2'= neutral/ don’t know, '1'= somewhat dissatisfied, '0'= very dissatisfied	1.22 (1.2)	0–4
Nature access	Agreement with sentence “I have access to enough natural land to meet all the needs of my household” | Ordinal | '4'= completely agree, '3'= somewhat agree, '2'= neutral/ don’t know, '1'= somewhat disagree, '0'= completely disagree	0.46 (1.01)	0–4
Education	Highest education level completed | Ordinal | ‘6’= university, '5'= college, '4'= secondary (form 1-6), '3'= primary (standard 5-7), '2'= primary (standard 1-4), '1'= no formal education but can read and write, '0'= no formal education	2.6 (1.21)	0–5
Overall quality of life	Level of life satisfaction | Ordinal | ‘0’= not at all satisfied to ‘10’= completely satisfied	4.11 (2.86)	0–10

### Well-being composite indicator

Following the approach developed by Loveridge et al. (2020), we used 20 indicators along five well-being dimensions to calculate a well-being composite indicator. The indicators used are described in Table 2. These indicators are representative of the five well-being domains put forward in the Millennium Ecosystems Assessment (MEA 2003), namely ‘basic material for a good life’, ‘health’, ‘social relations’, ‘security’, and ‘freedom of choice and action’. They were selected through the Well-being Indicator Selection Protocol (Loveridge et al. 2020), except diet diversity, which was added posteriorly for a more balanced representation of food security in the index. Prior to combining the variables, the data was checked for missing values and normalized. Missing cases were assumed to be missing at random and were deleted. Variables where higher values meant a more negative outcome were inverted. Normalization was achieved by dividing each variable by its respective maximum. To calculate the index, each variable was weighted in relation to the number of variables within the corresponding dimension, ensuring that all dimensions carried the same weight in the final composite index, irrespective of the number of constituting variables.

### Data analysis

After excluding 47 respondents through quality control (survey categorized as poor quality by the interviewer; respondents lived in region for less than 1 year; respondents stated no participation in farming activities or decisions), analysis was conducted on the resulting 420 valid questionnaires. The sample had adequate gender balance (200 adult men and 220 adult women). We used linear mixed effects models fitted by maximum likelihood. The fixed effects in each model are described in Table 1. All three models include village as the random effect, as we expected values within each village to be more similar than values between villages (Harrison et al. 2018). Interviewer was added in all models as a control fixed effect, rather than a random effect, due to its small number of levels (*n*=3) (Bolker et al. 2009).

The response variables for model 1 (agroecological intensity) and model 2 (staple crop yield) were log transformed, due to their right-skewed distribution. The correlation coefficients between model covariates were low. We acknowledge the high correlation between well-being indicator ‘land area’ and model covariate ‘plot ownership’ (rho=0.82, *p*-value<0.001). However, we decided to maintain ‘plot ownership’ as covariate in model 3 for consistency between the three models, due to the low correlation between ‘plot ownership’ and the response (well-being) in model 3 (rho= 0.15, *p*-value = 0.002), and the lack of significant differences based on a chi-square test between models with and without that covariate (chisq=2.00, *p*-value=0.157). Visual inspection of residual plots did not indicate deviations from homoscedasticity and normality, with the exception of model 2 where moderate deviations were observed.

Models were fitted with R package ‘lme4’. The Satterthwaite’s method was used to approximate degrees of freedom and calculate *p*-values (R package 'lmerTest'; Kuznetsova et al. 2017). Both the marginal (representing variance explained by the fixed effects only) and conditional Pseudo-R-squared values (assessing the variance explained by the entire model with fixed and random effects) were calculated using ‘MuMIn’ R package (Nakagawa et al. 2017). Confidence intervals were computed using likelihood ratio tests. We focus on full/global models as we are interested in the relationships between all covariates. The use of full models has been advocated in the literature as a valid alternative to the shortcomings of stepwise deletion and best fit models (Whittingham et al. 2006; Harrison et al. 2018; Smith 2018). Furthermore, our full models are not over-parameterized using the rule of thumb of a minimum of 10 observations for each parameter (Harrison et al. 2018). Non-parametric *W* and chi-squared statistics were calculated with Wilcoxon rank sum and Kruskal-Wallis tests, respectively, to assess significant differences in the distribution of two or more than two samples. Tukey’s ‘Honest Significant Difference’ (HSD) post hoc test complemented the Kruskal-Wallis tests by calculating pairwise comparisons of the mean between multiple groups. We used R package ‘ggplot2’ to create plots (Wickham 2016) and QGIS to create the map in Fig. 2.

## Results

We start by reporting on the variables described in Tables 1 and 2 and their associations, followed by the results of our three-tiered modelling approach. Overall, respondents reported an average well-being index of 0.546 (standard deviation= 0.165). The composite indicator had a normal distribution. Mean village well-being ranged from 0.420 (SD= 0.167) to 0.625 (SD= 0.185). Of the five dimensions that contribute to the well-being metric (Table 2), average values were lowest for the freedom dimension and highest for social relations.

Most farmers applied at least one agroecological practice in their farms (61.9%). The most common agroecological practices were mulching (*n*= 123), intercropping (*n*= 103), and post-harvest use of residues (*n*= 71). Farmers that applied at least one agroecological practice on average owned more land (5.11 vs 4.00 acres; *W*= 17858, *p*-value= 0.013) and listed a higher number of activities contributing to their household livelihood (*W*= 14250, *p*-value< 0.001). Education levels were similar regardless of applying agroecological practices or not (*W*= 19837, *p*-value= 0.356). Of the individual well-being indicators, those more highly correlated with agroecological intensity were number of livelihood-generating activities pursued (rho= 0.35, *p*-value< 0.001), perception of access to nature (rho= 0.26, *p*-value< 0.001), and most valuable livestock owned (rho= 0.26, *p*-value< 0.001) (Table 2). Results suggest that the use of agroecological practices contributes to farmer well-being along multiple dimensions, with significant improvements for the material (*W* = 18272, *p*-value = 0.036) and security (*W* = 17628, *p*-value= 0.009) dimensions.

**Fig. 3:**
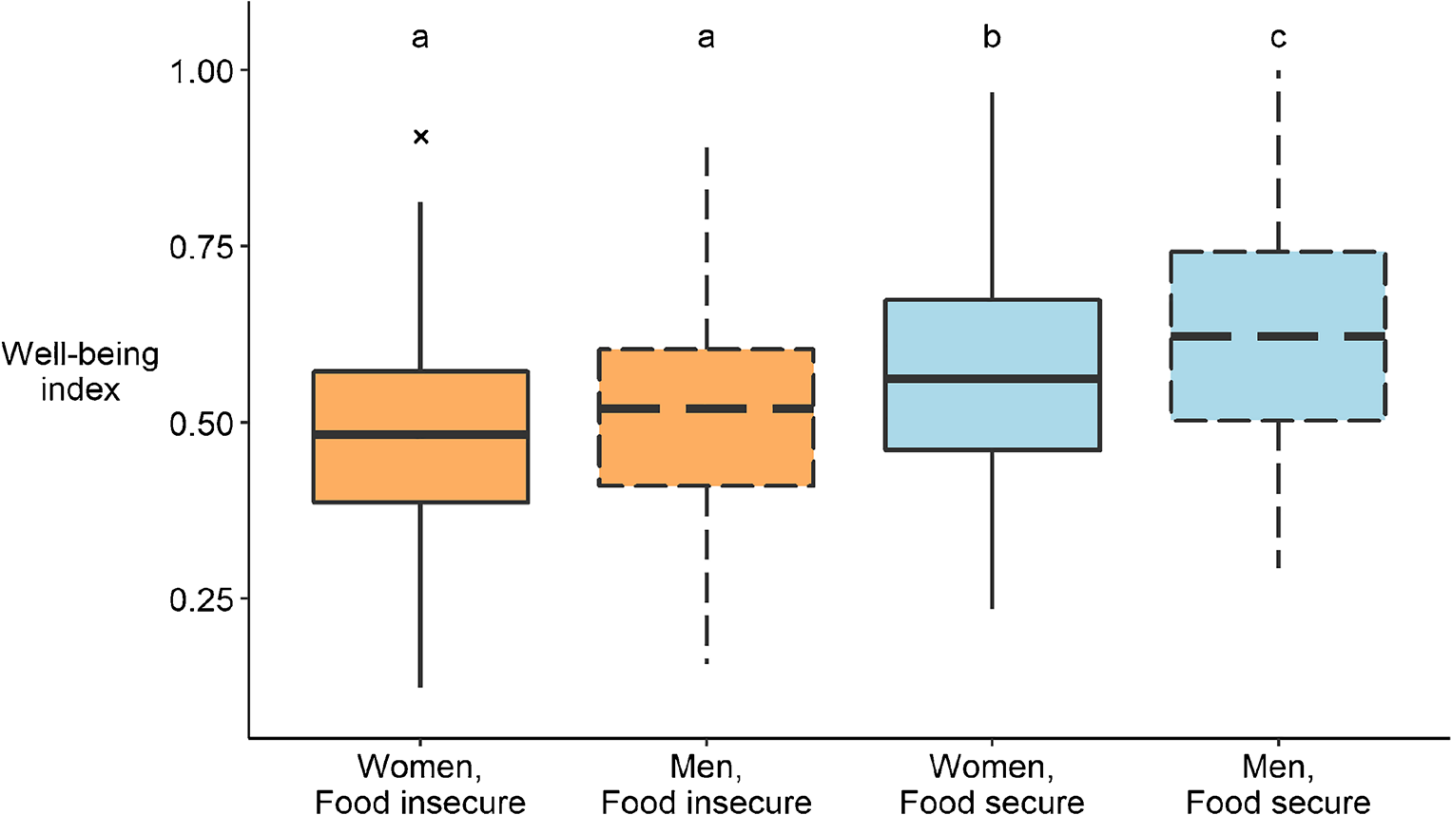
Boxplot comparing the distribution of the well-being indicator for four different groups: food insecure men (orange, dashed line), food-secure men (blue, dashed line), food-insecure women (orange, solid line), and food-secure women (blue, solid line). Food security is defined using the ‘Food needs’ variable described in Table 1. Respondents that always or often had problems satisfying the food needs of the household were considered food insecure. The middle line shows the median, the box defines the interquartile range, and the whiskers extend to 1.5 times the interquartile range. Outliers are pictured as crosses. The letters above the boxplots refer to the results of Tukey’s HSD post hoc test

Women with higher food needs had lower mean well-being (chisq= 46.87, *p*-value< 0.001), although not significantly different from men with high food needs (Fig. 3). In fact, 50.5% of respondents have been food insecure at least sometimes over the previous year. Female respondents reported similarly sized farms and levels of plot ownership, but on average owned a lower number of plots than men (1.66 vs 2.18; *W* = 11884, *p*-value = 0.029). They were less likely to have received farming advice in the last 3 years (*W*= 24410, *p*-value= 0.014) and to have used different categories of synthetic inputs (*W*= 25682, *p*-value= 0.002). Women also had a lower share of their land dedicated to cash crops than men (*W*= 24141, *p*-value= 0.015) and were less likely to agree that natural areas are good for their livelihoods (*W*= 25476, *p*-value= 0.003) and that environmental conditions would improve over the next 5 years (*W*= 24546, *p*-value= 0.030).

Unsurprisingly, landowners were much more likely to consider that they have access to enough fertile land to meet all the needs of their household (*W*= 8593.5, *p*-value< 0.001). They also had more trees planted in their farms (*W*= 12694, *p*-value< 0.001) and more tree richness with a mean of 1.76 in-farm tree species for landowners versus 0.97 tree species for other tenure arrangements (*W*= 13196, *p*-value< 0.001). In fact, not being the landowner was the second main reason (*n*= 133 respondents) given for not planting more trees, after small farm size (*n*= 151), and before concerns that tree shade reduces crop yield (*n*= 128).

Loss of crop production to animals, both invertebrate and vertebrate, is a considerable issue in the study area, with 61.9% of respondents stating that they lost more than a 1/4 of their production to animals. Levels of invertebrate crop damage are significantly higher in farms that use pesticides (*W*= 15280, *p*-value< 0.001) but similar between farms that use none versus at least one agroecological practice. Perceived crop damage caused by invertebrates is on average higher than the damage caused by vertebrates. However, the 95 respondents that indicated elephants as the main cause of vertebrate damage stated a considerably higher perceived damage when compared with farmers experiencing damage mainly from other vertebrates (2.28 vs 0.90, *W*= 128045, *p*-value< 0.001). Farmers experiencing elephant crop damage also had a significantly more negative perception of the impact nature has on their livelihood, regardless of the level of damage due to invertebrates (chisq= 15.68, *p*-value= 0.001).

**Fig. 4:**
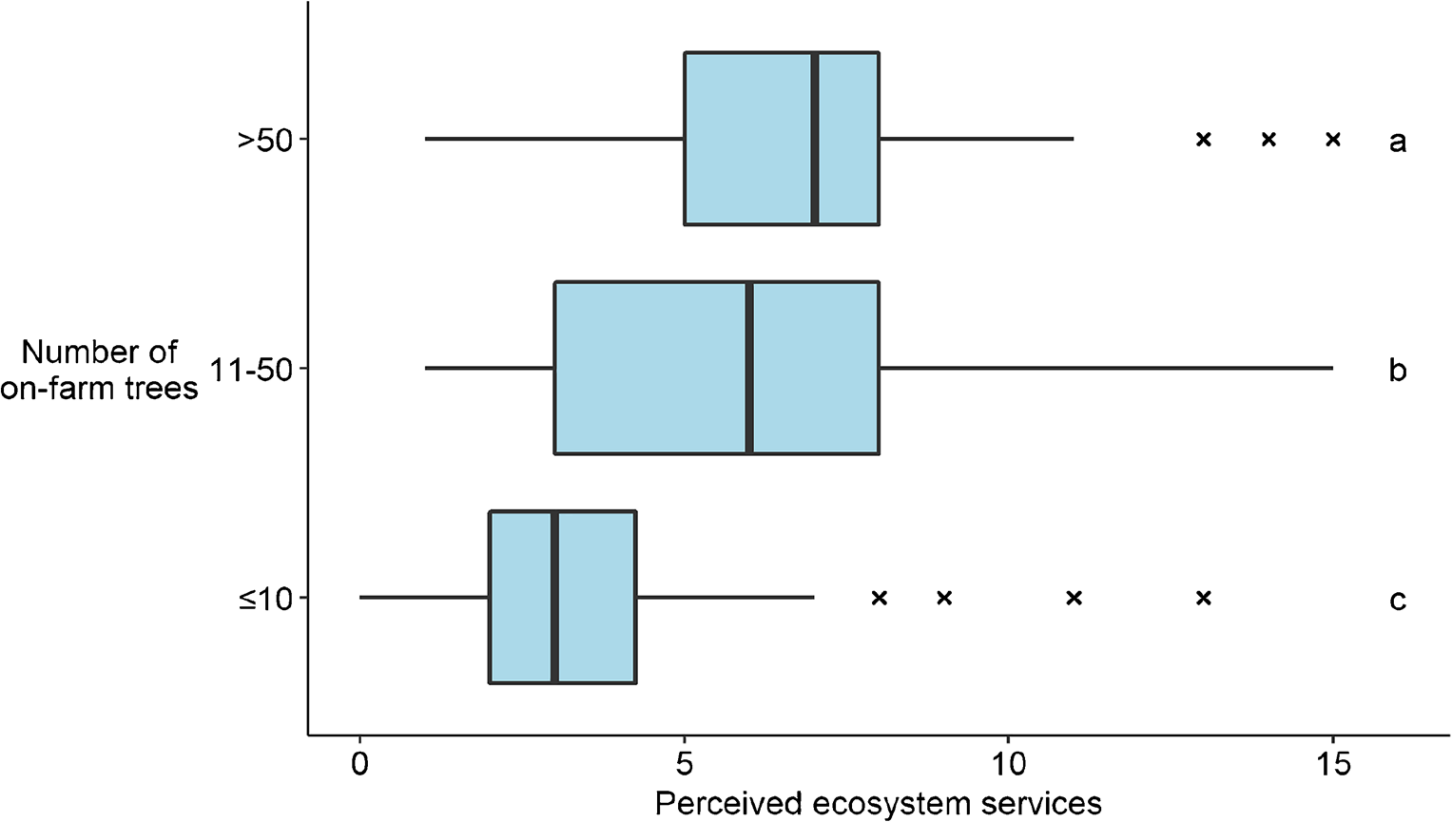
Boxplot relating the total number of ecosystem services perceived by the respondent as being provided by natural habitats in and around the farm and the number of trees planted in their farm. The middle line shows the median, the box defines the interquartile range, and the whiskers extend to 1.5 times the interquartile range. Outliers are pictured as crosses. The letters above the boxplots refer to the results of Tukey’s HSD post hoc test

Slightly less than a third of respondents (29.8%) reported receiving agricultural advice in the last 3 years. Main sources of advice were the extension service (*n*= 68), non-governmental organizations (*n*= 22), and cooperatives (*n*= 16). Farmers that received training were more likely to take measures to protect their crops against wildlife (*W*= 16022, *p*-value = 0.009), for example. Training was also positively correlated with the use of different synthetic inputs (rho= 0.190, *p*-value< 0.001), the use of pesticide specifically (rho= 0.157, *p*-value= 0.001), and with agroecological intensity (rho= 0.198, *p*-value< 0.001). The association between farming advice and the number of trees planted in-farm was relatively weaker (rho= 0.102, *p*-value= 0.036). Trained farmers were not more likely to have planted at least one tree in the previous year, nor more likely to be landowners. There was an association between farming training and perception of the number of ecosystem services being provided by nature (rho= 0.2090, *p*-value< 0.001). That perception was strongly correlated with the number of trees in-farm (rho= 0.440, *p*-value< 0.001) (Fig. 4). The correlation between number of in-farm trees and agroecological intensity was also positive (rho= 0.264, *p*-value< 0.001).

Advancing to the results of our modelling approach (Table 3), models 1 and 3 show that agroecological intensity and human well-being are influenced by different variables in our study area. Model 2, with staple crop yield as response, only had one significant covariate (agroecological intensity) and was poorly fitted (conditional R^2^= 0.122). Women farmers were more likely to use more agroecological practices in their farms. A higher awareness of ecosystem service provision also had a positive effect on agroecological intensity, as well as having received farming training, using more synthetic inputs, and being landowner. In model 3, variables representing socioeconomic conditions, perceptions, and farming practices all were found to have an impact on the multidimensional human well-being composite indicator. Gender and age were significant variables, with younger and male farmers showing higher well-being levels. Group membership and local-level social responsibilities were also positively linked with well-being. As expected, that was also the case for lower food needs. Interestingly, having positive perceptions of the impact of nature on their livelihood also contributed to farmer well-being. When adjusted by the other covariates, land ownership and perceived damage to crops were not significant drivers of well-being in our study area. Finally, higher number of different synthetic inputs and higher in-farm agroecological intensity both also contributed to well-being.

**Table 3:** Modelling coefficients (Coefs), 95% confidence intervals (CI), and *p*-values. Response variables are agroecological intensity for model 1, staple crop yield for model 2, and human well-being for model 3. Significant *p*-values are in bold

Variables	Model 1 Coefs	Model 1 CI	Model 1 *P*-values	Model 2 Coefs	Model 2 CI	Model 2 *P*-values	Model 3 Coefs	Model 3 CI	Model 3 *P*-values
Intercept	−0.0439	−0.515 — 0.412	0.8503	0.1009	0.014 — 0.189	**0.0236**	0.3105	0.218 — 0.403	**0**
Age	0.0008	−0.003 — 0.005	0.7079	−0.0001	−0.001 — 0.001	0.8571	−0.0012	−0.002 — 0	**0.0164**
Farming advice	0.1307	0.005 — 0.256	**0.0408**	−0.0044	−0.032 — 0.023	0.7506	0.0203	−0.009 — 0.049	0.1724
Food needs	0.0086	−0.04 — 0.057	0.7246	0.0002	−0.01 — 0.01	0.9772	0.0377	0.027 — 0.049	**0**
Gender	0.1322	0.015 — 0.249	**0.027**	−0.0105	−0.036 — 0.015	0.4116	−0.0295	−0.057 — −0.002	**0.033**
Group membership	0.023	−0.022 — 0.068	0.3197	−0.0088	−0.019 — 0.001	0.0765	0.032	0.021 — 0.043	**0**
Perceived crop damage	−0.0504	−0.169 — 0.068	0.405	−0.0067	−0.032 — 0.019	0.6079	−0.0125	−0.04 — 0.015	0.3711
Perceived ecosystem services	0.0314	0.012 — 0.051	**0.0015**	0.0021	−0.002 — 0.006	0.3293	0.0021	−0.002 — 0.007	0.3709
Perceived nature impact on livelihood	0.044	−0.007 — 0.095	0.0916	0.0053	−0.006 — 0.016	0.3402	0.0126	0.001 — 0.024	**0.0341**
Perceived future conditions	0.0031	−0.066 — 0.073	0.9291	−0.0084	−0.023 — 0.007	0.2704	0.0088	−0.007 — 0.025	0.28
Plot ownership	0.2172	0.088 — 0.346	**0.001**	−0.0251	−0.053 — 0.003	0.08	0.0218	−0.008 — 0.052	0.1572
Synthetic inputs	0.0661	0.014 — 0.118	**0.0122**	0.0068	−0.004 — 0.018	0.2327	0.0167	0.005 — 0.029	**0.0062**
Village responsibilities	0.1038	−0.031 — 0.238	0.1304	0.0096	−0.019 — 0.039	0.5171	0.0587	0.028 — 0.09	**0.0002**
Agroecological intensity	NA	NA	NA	0.0102	0.003 — 0.017	**0.0056**	0.0089	0.001 — 0.017	**0.0234**
Staple crop yield	NA	NA	NA	NA	NA	NA	0.0092	−0.065 — 0.084	0.8075
Marginal R2		0.175			0.065			0.390	
Conditional R2		0.371			0.122			0.411	

## Discussion

Understanding interlinkages between agroecology, food security, and human well-being in tropical rural Africa is crucial to improve the effectiveness of agricultural development policies and programs. Our results identify multiple factors, namely indicators of socioeconomic conditions, perceptions of benefits from nature, and farming practices, with an impact on agroecological intensity at farm-level. Our findings also show that higher agroecological intensity at farm-level can contribute positively to the food security and well-being of smallholder farmers. There is limited empirical evidence in the literature for the relationship between agroecological intensity and smallholder farmer well-being in rural landscapes in the tropics (Miller et al. 2020). Still, our results align with previous studies indicating that, despite high variability, the overall impact of sustainable agricultural practices on livelihoods and well-being in the tropics tends to be positive (Reed et al. 2017; Mdee et al. 2018; Leakey 2020; Castle et al. 2021).

Our analysis suggests that the use of agroecological practices improves farmer well-being along multiple dimensions, with significant improvements for the material and security dimensions. Younger or male farmers, farmers with a more positive perception of how nature impacts their livelihood, farmers that had village responsibilities or were involved in local groups and had lower food needs, and farmers applying more different types of synthetic inputs were all more likely to have higher well-being. The observed relationships suggest that greater access to social, produced, and natural capital within a community constitutes an advantage that is reflected on the well-being of individuals (Isham 2002; Jeckoniah et al. 2020; Dasgupta 2021). Interestingly, women were more likely to have higher agroecological intensity in their farms, in contrast to previous findings (Miller et al. 2017), but that does not seem to improve their well-being. Mean well-being of women with a civil status other than married was similar to those of men in the same situation. But, for married respondents, well-being is significantly higher for men (0.582 vs 0.537; *W*= 10817, *p*-value= 0.015). This indicates that, despite women being more likely to engage with different agroecological practices, married men seem more likely to benefit from improvements to well-being generated by those practices (Masamha et al. 2018). Exploring the social dynamics that might be behind this result is beyond the scope of this study.

Reassuringly, in light of recent discussions on sustainable intensification of agriculture, our results suggest that higher agroecological intensification increases yield of staple crops. This association is highly variable in the literature. For example, a recent review failed to find significant effect of agroforestry interventions on yields, although it found a neutral to positive impact on nutrition and food security (Castle et al. 2021). Food security for smallholder farmers in the tropics is highly linked to staple crop yield (IFAD and UNEP 2013). Agroforestry can work as a safety net for food-insecure households (Ndoli et al. 2021). Still, the low goodness of fit of model 2 indicates that most of the variation is not being captured by the model covariates, so it is likely that important drivers of staple crop yield are missing. These might include further socioeconomic factors, external drivers, such as market or regulatory factors, or environmental variables, such as soil fertility or distance to water resources (Milheiras et al. 2022).

The use of agroecological practices, such as mulching, was relatively common in our sample, which indicates that at least some of those practices are relatively accessible and carry low short-term investment risks (Jerneck and Olsson 2014). Being female, having a higher awareness of ecosystem service provision, having received farming advice in the previous 3 years, being the landowner and applying different synthetic inputs were variables that significantly contributed to a higher uptake of agroecological practices. Land ownership has previously been identified as a driver of sustainable agricultural practices (Kassie et al. 2013; Teklewold et al. 2013; Miller et al. 2017). Women might be applying more agroecological practices in our sample as a consequence of their choices on crop type, with a stronger preference for planting staple crops than men, or as result of more limited access to productive resources (Masamha et al. 2018; Mason et al. 2015). But further research is needed to investigate the causal mechanism in this relationship. Our results also suggest that farmers that are more aware of ecosystem service provision adapt their practices accordingly in favor of practices that protect future benefits, which is in line with previous research (Meijer et al. 2015; Piñeiro et al. 2020). Positive perceptions on nature can be reinforced and negative ones mitigated to favor nature conservation outcomes (Sanou et al. 2019). It has been shown, and our study provides additional evidence, that providing extension services and training to farmers are effective ways of promoting sustainable agricultural practices, especially when also acknowledging farmer perceptions of future benefits and predicted trade-offs between economic, environmental, and social outcomes (Meijer et al. 2015; Sanou et al. 2019; Piñeiro et al. 2020). There is strong evidence that measures that increase plant diversity in agroecosystems can increase crop and forage yield, wood production, yield stability, pollinators, weed suppression, and pest suppression (Isbell et al. 2017). This information needs to be translated into clear, straightforward, locally adjusted messages targeted at smallholder farmers and other stakeholders.

It is interesting to note that the amount of crop damage perceived by farmers was not a significant driver of agroecological intensification, nor well-being. This suggests that current levels of damage are expected, localized, and/or not intense enough to have a direct influence on well-being. Still, elephant damage seems to sharply change farmer perceptions on how beneficial nature is to their livelihood, and this will indirectly affect well-being. Elephant damage might also considerably reduce local support for conservation interventions (Matejcek and Verne 2021). And we know that the uptake of sustainable agricultural practices is reduced if those practices are perceived to attract or shelter species considered to be problematic (Pfund et al. 2011). If ongoing conversion rates of natural habitat to cropland continue (Munishi and Jewitt 2019), these conflicts, and local animosity towards wildlife, are likely to be aggravated.

The use of more types of synthetic input is associated with higher agroecological intensity in our data, which suggests smallholder farmers are combining practices to achieve complementary farming goals, namely, higher farm resilience and farm productivity. In a way, this reflects the regulatory context in the country, where the policy that frames agricultural development (Agricultural Sector Development Program, phase II) prioritizes both sustainable land use management (component 1) and enhanced productivity and profitability (component 2). Also in SAGCOT plans for the region, despite their focus on large-scale commercial farms, there is a strategy for sustainable intensification targeted at smallholder farmers (EcoAgriculture Partners 2012). While ecological intensification is presented in that strategy as part of the solution, too little detail is provided on how farmers should navigate any eventual trade-offs between industrial and ecological intensification practices. It is relevant to understand how both approaches affect well-being, inclusively when both are being simultaneously implemented at farm or landscape scale. For now, we have an incomplete picture of the impacts of sustainable agricultural practices on food production and well-being over a wide range of farming systems (Reed et al. 2017; Castle et al. 2021). Policymakers should not assume that industrial farming intensification will inevitably result in higher human well-being, if that intensification is done at the expense of natural areas (Rasmussen et al. 2018) and/or human health (de Bon et al. 2014). Future research should focus on detailing trade-offs and synergies adjusted to local contexts, including on outcomes (e.g., profitability, food security) that are most relevant to smallholder farmers and specifically on how vulnerable groups are affected (Below et al. 2012; Kleijn et al. 2019; Castle et al. 2021). Moving forward from our observational study, we recommend measuring the impact on well-being of specific interventions linked to sustainable agricultural practices using quasi-experimental approaches (Miller et al. 2020).

Our analysis should be interpreted in the context of our case study and we advise against generalizations to broader spatial scales or dissimilar socioecological systems. Furthermore, our approach comes with at least three possible limitations. First, we have not included direct metrics of wealth and education levels as model covariates, due to those variables being part of the well-being index, although they have been associated with both agroecological intensity and well-being (Miller et al. 2020). However, it can be argued that other covariates indirectly capture the wealth of individual respondents (for example the food needs variable) and the respondents’ education level is strongly correlated with age (rho= −0.33, *p*-value< 0.001) and village responsibilities (rho= 0.31, *p*-value< 0.001). Second, while intra-household decision-making dynamics can result in household members not necessarily sharing the same views, the use of individual-based variables to analyze farming practices requires caution, as most farming decisions will be done in the context of a household, a family, and a local community (Anderson et al. 2017). Third, we could not include total area of land owned as a model covariate, since it is one of the indicators used to calculate well-being, but exploratory analysis suggests that using it instead of the binary of plot ownership variable would not change results substantially if it had substituted the binary plot ownership variable used.

## Conclusions

This study presents new empirical evidence in support of the well-being benefits to smallholder farmers of the implementation of agroecological practices. We show that practices taking advantage of nature’s contributions to people within agricultural systems can contribute positively to food security and human well-being of smallholder farmers in rural landscapes of the tropics. In addition to the positive relationship between agroecological practices and farmer well-being, other conclusions are noted. First, our research finds that farmers applying agroecological practices continue to use conventional practices too, and both are contributing to higher well-being in our study area. This suggests that a transition to more ecological farming can have impact on human well-being, even if that transition complements rather than fully replaces conventional farming. Second, our results corroborate previous studies on how fundamental technical training and capacity building of smallholder farmers is for the uptake of sustainable agricultural practices. That uptake will be more successful if institutions promoting it are able to show how farmers will benefit, via extension services or demonstration farms, for example. Finally, our study confirms that well-being metrics are a valuable tool for measuring, in a comprehensive way, the impacts of farming practices and policy interventions at local scales. Understanding which factors increase individual well-being will allow more effective policies across sectors. The challenge in our study area, and in similar landscapes, is to find the incentives and interventions that maximize the benefits from agriculture to human well-being by integrating, rather than opposing, food production and nature conservation goals. More research is needed to understand which combination of agricultural practices best contribute to well-being under specific environmental, social, and economic conditions. This study informs the design of nature-positive interventions, in the context of agricultural development and land use management, aiming to improve the well-being of rural communities in sub-Saharan Africa.

## Data Availability

The datasets generated and analyzed during the current study are available in the Newcastle University data repository at 10.25405/data.ncl.17192867.

## Code availability

The code used for data analysis is available upon request to the corresponding author.

## References

[CR1] Agarwala M, Atkinson G, Fry BP, Homewood K, Mourato S, Rowcliffe JM, Wallace G, Milner-Gulland EJ (2014). Assessing the relationship between human well-being and ecosystem services: a review of frameworks. Conserv Soc.

[CR2] Akinnifesi FK, Ajayi OC, Sileshi G, Chirwa PW, Chianu J (2010). Fertiliser trees for sustainable food security in the maize-based production systems of East and Southern Africa. A review. Agron Sustain Dev.

[CR3] Altieri MA, Funes-Monzote FR, Petersen P (2012). Agroecologically efficient agricultural systems for smallholder farmers: contributions to food sovereignty. Agron Sustain Dev.

[CR4] Anderson CL, Reynolds TW, Gugerty MK (2017). Husband and wife perspectives on farm household decision-making authority and evidence on intra-household accord in rural Tanzania. World Dev.

[CR5] Bashir MK, Schilizzi S (2013). Determinants of rural household food security: a comparative analysis of African and Asian studies. J Sci Food Agric.

[CR6] Beauchamp E, Woodhouse E, Clements T, Milner-Gulland EJ (2018). “Living a good life”: conceptualizations of well-being in a conservation context in Cambodia. Ecol Soc.

[CR7] Below TB, Mutabazi KD, Kirschke D, Franke C, Sieber S, Siebert R, Tscherning K (2012). Can farmers’ adaptation to climate change be explained by socio-economic household-level variables?. Glob Environ Chang.

[CR8] Bolker BM, Brooks ME, Clark CJ, Geange SW, Poulsen JR, Stevens MHH, White JSS (2009). Generalized linear mixed models: a practical guide for ecology and evolution. Trends Ecol Evol.

[CR9] Campbell BM, Beare DJ, Bennett EM, Hall-Spencer JM, Ingram JSI, Jaramillo F, Ortiz R, Ramankutty N, Sayer JA, Shindell D (2017). Agriculture production as a major driver of the earth system exceeding planetary boundaries. Ecol Soc.

[CR10] Caron P, Biénabe E, Hainzelin E (2014). Making transition towards ecological intensification of agriculture a reality: the gaps in and the role of scientific knowledge. Curr Opin Environ Sustain.

[CR11] Castle SE, Miller DC, Ordonez PJ, Baylis K, Hughes K (2021). The impacts of agroforestry interventions on agricultural productivity, ecosystem services, and human well-being in low- and middle-income countries: a systematic review. Campbell Syst Rev.

[CR12] CGIAR (Consultative Group on International Agricultural Research) (2015) Climate Change, Agriculture and Food Security (CCAFS) household baseline survey 2010-2012, Harvard Dataverse, 10.7910/DVN/IUJQZV

[CR13] Chappell MJ, LaValle LA (2009). Food security and biodiversity: can we have both? An agroecological analysis. Agric Hum Values.

[CR14] Costanza R, Daly L, Fioramonti L, Giovannini E, Kubiszewski I, Mortensen LF, Pickett KE, Ragnarsdottir KV, de Vogli R, Wilkinson R (2016). Modelling and measuring sustainable wellbeing in connection with the UN Sustainable Development Goals. Ecol Econ.

[CR15] Dasgupta P (2021) The economics of biodiversity: the Dasgupta review, vol 2021. HM Treasury, London

[CR16] de Bon H, Huat J, Parrot L, Sinzogan A, Martin T, Malézieux E, Vayssières JF (2014). Pesticide risks from fruit and vegetable pest management by small farmers in sub-Saharan Africa. A review. Agron Sustain Dev.

[CR17] de Lange E, Woodhouse E, Milner-Gulland EJ (2016). Approaches used to evaluate the social impacts of protected areas. Conserv Lett.

[CR18] Dinesen L (2016). Kilombero Valley Floodplain (Tanzania). In: The Wetland Book.

[CR19] Duriaux Chavarría JY, Baudron F, Sunderland T (2018). Retaining forests within agricultural landscapes as a pathway to sustainable intensification: evidence from Southern Ethiopia. Agric Ecosyst Environ.

[CR20] EcoAgriculture Partners (2012) The SAGCOT Greenprint - a green growth investment framework for the Southern Agricultural Growth Corridor of Tanzania. https://ecoagriculture.org/publication/a-vision-for-agriculture-green-growth-in-the-southern-agriculture-growth-corridor-of-tanzania-sagcot/greenprint-for-the-southern-agricultural-growth-corridor-of-tanzania-sagcot/

[CR21] EDI (Economic Development Initiatives) (2007). Tanzania Core Welfare Indicators Questionnaire Survey 2006-2007.

[CR22] Enns C (2018). Mobilizing research on Africa’s development corridors. Geoforum.

[CR23] ESA (2017). ESA Climate Change Initiative - Land Cover project 2017. S2 prototype LC 20m map of Africa 2016

[CR24] FAO (2017) The future of food and agriculture - trends and challenges. Food and Agriculture Organization of the United Nations, Rome

[CR25] Frongillo EA, Nguyen HT, Smith MD, Coleman-Jensen A (2017). Food insecurity is associated with subjective well-being among individuals from 138 countries in the 2014 gallup world poll. J Nutr.

[CR26] Harrison XA, Donaldson L, Correa-Cano ME, Evans J, Fisher DN, Goodwin CED, Robinson BS, Hodgson DJ, Inger R (2018). A brief introduction to mixed effects modelling and multi-model inference in ecology. PeerJ.

[CR27] Hartig T, Mitchell R, De Vries S, Frumkin H (2014). Nature and health. Annu Rev Public Health.

[CR28] Hartung C, Anokwa Y, Brunette W et al (2010) Open data kit: tools to build information services for developing regions. In: Proceedings of the 4th ACM/IEEE international conference on information and communication technologies and development, vol 18, pp 1–12. 10.1145/2369220.2369236

[CR29] HLPE (2019) Agroecological and other innovative approaches for sustainable agriculture and food systems that enhance food security and nutrition. In: A report by the high level panel of experts on food security and nutrition of the committee on world food security, Rome

[CR30] Hughes K, Morgan S, Baylis K, Oduol J, Smith-Dumont E, Vågen TG, Kegode H (2020). Assessing the downstream socioeconomic impacts of agroforestry in Kenya. World Dev.

[CR31] IFAD and UNEP (2013) Smallholders, food security and the environment. International Fund for Agricultural Development, Rome

[CR32] Isbell F, Adler PR, Eisenhauer N, Fornara D, Kimmel K, Kremen C, Letourneau DK, Liebman M, Polley HW, Quijas S, Scherer-Lorenzen M (2017). Benefits of increasing plant diversity in sustainable agroecosystems. J Ecol.

[CR33] Isham J (2002). The effect of social capital on fertiliser adoption: evidence from rural Tanzania. J Afr Econ.

[CR34] Jeckoniah JN, Akyoo EP, Kabote S (2020). Large scale agricultural investments and its impact on gender relations and wellbeing of small holder farmers: evidence from Kilombero Valley in Tanzania. African J L Policy Geospatial Sci.

[CR35] Jerneck A, Olsson L (2014). Food first! Theorising assets and actors in agroforestry: risk evaders, opportunity seekers and “the food imperative” in sub-Saharan Africa. Int J Agric Sustain.

[CR36] Jones AD (2017). Critical review of the emerging research evidence on agricultural biodiversity, diet diversity, and nutritional status in low- and middle-income countries. Nutr Rev.

[CR37] Kassie M, Jaleta M, Shiferaw B et al (2013) Adoption of interrelated sustainable agricultural practices in smallholder systems: evidence from rural Tanzania. Technol Forecast Soc Change:80. 10.1016/j.techfore.2012.08.007

[CR38] Kleijn D, Bommarco R, Fijen TPM, Garibaldi LA, Potts SG, van der Putten WH (2019). Ecological intensification: bridging the gap between science and practice. Trends Ecol Evol.

[CR39] Kremen C, Merenlender AM (2018). Landscapes that work for biodiversity and people. Science.

[CR40] Kremen C, Miles A (2012). Ecosystem services in biologically diversified versus conventional farming systems: benefits, externalities, and trade-offs. Ecol Soc.

[CR41] Kuznetsova A, Brockhoff PB, Christensen RHB (2017) lmerTest Package: Tests in Linear Mixed Effects Models. J Stat Softw 82. 10.18637/jss.v082.i13

[CR42] Laurance WF, Sloan S, Weng L, Sayer JA (2015). Estimating the environmental costs of Africa’s massive “Development Corridors”. Curr Biol.

[CR43] Leakey RRB (2020). A re-boot of tropical agriculture benefits food production, rural economies, health, social justice and the environment. Nat Food.

[CR44] Loveridge R, Sallu SM, Pesha IJ, Marshall AR (2020). Measuring human wellbeing: a protocol for selecting local indicators. Environ Sci Policy.

[CR45] Masamha B, Thebe V, Uzokwe VNE (2018). Mapping cassava food value chains in Tanzania’s smallholder farming sector: the implications of intra-household gender dynamics. J Rural Stud.

[CR46] Mason R, Ndlovu P, Parkins JR, Luckert MK (2015). Determinants of food security in Tanzania: gendered dimensions of household headship and control of resources. Agric Hum Values.

[CR47] Matejcek A, Verne J (2021). Restoration-as-development? Contesting aspirational politics regarding the restoration of wildlife corridors in the Kilombero Valley, Tanzania. Eur J Dev Res.

[CR48] Mbow C, van Noordwijk M, Prabhu R, Simons T (2014). Knowledge gaps and research needs concerning agroforestry’s contribution to sustainable development goals in Africa. Curr Opin Environ Sustain.

[CR49] Mdee A, Wostry A, Coulson A, Maro J (2018). A pathway to inclusive sustainable intensification in agriculture? Assessing evidence on the application of agroecology in Tanzania. Agroecol Sustain Food Syst.

[CR50] MEA (2003) Millennium ecosystem assessment: ecosystems and human well-being - a framework for assessment. Island Press, Washington

[CR51] Meijer SS, Catacutan D, Ajayi OC, Sileshi GW, Nieuwenhuis M (2015). The role of knowledge, attitudes and perceptions in the uptake of agricultural and agroforestry innovations among smallholder farmers in sub-Saharan Africa. Int J Agric Sustain.

[CR52] Milheiras SG, Sallu SM, Marshall AR, Shirima DD, Kioko EN, Loveridge R, Moore E, Olivier P, Teh YA, Rushton S, Pfeifer M (2022) A framework to assess forest-agricultural landscape management for socioecological well-being outcomes. Front For Glob Change 5:709971. 10.3389/ffgc.2022.709971

[CR53] Miller DC, Muñoz-Mora JC, Christiaensen L (2017). Prevalence, economic contribution, and determinants of trees on farms across Sub-Saharan Africa. For Policy Econ.

[CR54] Miller DC, Ordoñez PJ, Brown SE, Forrest S, Nava NJ, Hughes K, Baylis K (2020) The impacts of agroforestry on agricultural productivity, ecosystem services, and human well-being in low-and middle-income countries: an evidence and gap map. Campbell Syst Rev 16. 10.1002/cl2.1066 10.1002/cl2.1066 PMC8356334 37131981

[CR55] Milner-Gulland EJ, Mcgregor JA, Agarwala M (2014). Accounting for the impact of conservation on human well-being. Conserv Biol.

[CR56] Morton JF (2007). The impact of climate change on smallholder and subsistence agriculture. Proc Natl Acad Sci USA.

[CR57] Munishi S, Jewitt G (2019). Degradation of Kilombero Valley Ramsar wetlands in Tanzania. Phys Chem Earth.

[CR58] Naeem S, Chazdon R, Duffy JE, Prager C, Worm B (2016). Biodiversity and human well-being: an essential link for sustainable development. Proc R Soc London B Biol Sci.

[CR59] Nakagawa S, Johnson PCD, Schielzeth H (2017). The coefficient of determination R2 and intra-class correlation coefficient from generalized linear mixed-effects models revisited and expanded. J R Soc Interface.

[CR60] National Bureau of Statistics (2013) Tanzania population and housing census 2012. Ministry of Finance, Dar es Salaam, and Office of Chief Government Statistician, Zanzibar

[CR61] Ndoli A, Mukuralinda A, Schut AG (2021). On-farm trees are a safety net for the poorest households rather than a major contributor to food security in Rwanda. Food Secur.

[CR62] Nijbroek RP, Andelman SJ (2016). Regional suitability for agricultural intensification: a spatial analysis of the Southern Agricultural Growth Corridor of Tanzania. Int J Agric Sustain.

[CR63] Ojedokun CA, Ugege BH, Kolade RI, Tunde-Francis AA, Odediran FA (2020). Contribution of agroforestry to farmers wellbeing in Forest Enclave, Edo State, Nigeria. J Appl Sci Environ Manag.

[CR64] Pfund J-L, Watts JD, Boissière M, Boucard A, Bullock RM, Ekadinata A, Dewi S, Feintrenie L, Levang P, Rantala S, Sheil D, Sunderland TCH, Urech ZL (2011). Understanding and integrating local perceptions of trees and forests into incentives for sustainable landscape management. Environ Manage.

[CR65] Piñeiro V, Arias J, Dürr J, Elverdin P, Ibáñez AM, Kinengyere A, Opazo CM, Owoo N, Page JR, Prager SD, Torero M (2020). A scoping review on incentives for adoption of sustainable agricultural practices and their outcomes. Nat Sustain.

[CR66] Pretty J, Bharucha ZP (2014). Sustainable intensification in agricultural systems. Ann Bot.

[CR67] Rasmussen LV, Coolsaet B, Martin A, Mertz O, Pascual U, Corbera E, Dawson N, Fisher JA, Franks P, Ryan CM (2018). Social-ecological outcomes of agricultural intensification. Nat Sustain.

[CR68] Reed J, van Vianen J, Foli S, Clendenning J, Yang K, MacDonald M, Petrokofsky G, Padoch C, Sunderland T (2017). Trees for life: the ecosystem service contribution of trees to food production and livelihoods in the tropics. For Policy Econ.

[CR69] Reyes-García V, Babigumira R, Pyhälä A, Wunder S, Zorondo-Rodríguez F, Angelsen A (2016). Subjective wellbeing and income: empirical patterns in the rural developing world. J Happiness Stud.

[CR70] Rivera M, Knickel K, de los Rios I (2018). Rethinking the connections between agricultural change and rural prosperity: a discussion of insights derived from case studies in seven countries. J Rural Stud.

[CR71] SAGCOT (2011) Southern agricultural growth investment blueprint. https://sagcot.co.tz/images/documents/SAGCOT-Invest-Blueprint.pdf

[CR72] Sanou L, Savadogo P, Ezebilo EE, Thiombiano A (2019). Drivers of farmers’ decisions to adopt agroforestry: evidence from the Sudanian savanna zone. Burkina Faso. Renew Agric Food Syst.

[CR73] Smith G (2018) Step away from stepwise. J Big Data 5. 10.1186/s40537-018-0143-6

[CR74] Soga M, Gaston KJ (2016). Extinction of experience: the loss of human-nature interactions. Front. Ecol Environ.

[CR75] Stiglitz JE, Sen A, Fitoussi J-P (2009) The measurement of economic performance and social progress revisited: reflections and overview. Sciences Po publications 2009–33. Sciences Po, Paris

[CR76] Sulle E (2017). Social differentiation and the politics of land: sugar cane outgrowing in Kilombero, Tanzania. J South Afr Stud.

[CR77] Teklewold H, Kassie M, Shiferaw B (2013). Adoption of multiple sustainable agricultural practices in rural Ethiopia. J Agric Econ.

[CR78] UNEP-WCMC and IUCN (2021) Protected planet: the world database on protected areas (WDPA) and world database on other effective area-based conservation measures (WD-OECM) [online], Cambridge

[CR79] Van Ittersum MK, Van Bussel LGJ, Wolf J (2016). Can sub-Saharan Africa feed itself?. Proc Natl Acad Sci USA.

[CR80] Wezel A, Casagrande M, Celette F, Vian JF, Ferrer A, Peigné J (2014). Agroecological practices for sustainable agriculture. A review. Agron Sustain Dev.

[CR81] Wezel A, Herren BG, Kerr RB, Barrios E, Gonçalves ALR, Sinclair F (2020). Agroecological principles and elements and their implications for transitioning to sustainable food systems. A review. Agron Sustain Dev.

[CR82] Wezel A, Soboksa G, McClelland S, Delespesse F, Boissau A (2015). The blurred boundaries of ecological, sustainable, and agroecological intensification: a review. Agron Sustain Dev.

[CR83] Whittingham MJ, Stephens PA, Bradbury RB, Freckleton RP (2006). Why do we still use stepwise modelling in ecology and behaviour?. J Anim Ecol.

[CR84] Wickham H (2016). ggplot2: elegant graphics for data analysis.

[CR85] World Bank (2016). CGAP Smallholder Household Survey 2016. Data accessed at https://microdata.worldbank.org/index.php/catalog/2584/study-description.

[CR86] Zhang W, Ricketts TH, Kremen C, Carney K, Swinton SM (2007). Ecosystem services and dis-services to agriculture. Ecol Econ.

